# Suppression of mTORC1 activity in senescent Ras-transformed cells neither restores autophagy nor abrogates apoptotic death caused by inhibition of MEK/ERK kinases

**DOI:** 10.18632/aging.101686

**Published:** 2018-11-27

**Authors:** Elena Y. Kochetkova, Galina I. Blinova, Olga A. Bystrova, Marina G. Martynova, Valeriy A. Pospelov, Tatiana V. Pospelova

**Affiliations:** 1 Institute of Cytology, Russian Academy of Sciences, St-Petersburg 194064, Russia

**Keywords:** senescence, mitochondria damage, lysosomes, autophagy, mTOR, MEK/ERK, kinase inhibitors

## Abstract

Autophagy is conservative catabolic process that degrades organelles, in particular, mitochondria, and misfolded proteins within the lysosomes, thus maintaining cellular viability. Despite the close relationship between mitochondrial dysfunction and cellular senescence, it is unclear how mitochondria damage can induce autophagy in senescent cells. We show that MEK/ERK suppression induces mitochondria damage followed by apoptosis of senescent Ras-expressing cells. To understand the role of persistent mTORC1 signaling in breaking the cAMPK-induced autophagy caused by mitochondrial damage, we inhibited mTORС1 with low concentrations of pp242. mTORC1 suppression neither restores the AMPK-induced autophagy nor decreases the level of apoptosis upon MEK/ERK inhibition. We discovered the existence of an alternative autophagy-like way that partially increases the viability of senescent cells under suppressed mTORC1. The pp242-treated cells survive due to formation of the non-autophagous LC3-negative vacuoles, which contain the damaged mitochondria and lysosomes with the following excretion the content from the cell. MEK/ERK activity is required to implement this process in senescent cells. Senescent cells exhibit distinctive spatial distribution of organelles and proteins that provides uncoupling of final participants of autophagy. We show that this feature stops the process of cytoprotective autophagy in response to MEK/ERK suppression, thus allowing selective elimination of senescent Ras-expressing cells.

## Introduction

Senescent cells are characterized by cell cycle arrest, an increase in cell size and mitochondrial mass together with mitochondrial dysfunction [1]. Senescent cells exhibit a version of the AMPK-regulated autophagy yet retaining high activity of the mammalian Target of Rapamycin Complex 1 (mTORC1) that can potentially block mTOR-dependent autophagy. The mTOR is a conserved serine/threonine kinase that regulates protein translation by 5′-AMP- activated protein kinase (AMPK). When mTORC1 is active, it stimulates protein, nucleotide and lipid biogenesis, simultaneously inhibiting the catabolic process of autophagy [2]. Autophagy is initiated through the ULK1 complex, which is regulated both by AMPK and mTORC1, which phosphorylate and thereby stimulate or slow down the ULK1 kinase activity [3]. However, when mTORC1 activity is anyway inhibited, the complex is not able to inhibit ULK1 [4]. In turn, AMPK can directly phosphorylate ULK1 and Atg13 to stimulate autophagy in many cell lines. In addition, AMPK suppresses mTORC1 and by that activates autophagy through Ulk1 phosphorylation at Ser555 residue [5,6]. The balance between AMPK and mTORC1 is maintained by the lysosomes that serve as a platform for both kinases, integrating the external and internal signals [7]. The lysosomes act as a signaling hub, favoring either mTORC1 (through v-ATPase-Regulator-Rags complex) or AMPK (through v-ATPase-Ragulator-AXIN/LKB1 axis) [8]. Activity of lysosome membrane-bound mTORC1 is dependent on the localization of lysosomes. In nutrient-rich conditions, lysosomes localize at the periphery of the cell, bringing mTORC1 in the proximity with growth factor receptors and thus favoring its activation. Nutrient deprivation leads to relocalization of lysosomes to the perinuclear region, where mTORC1 is inhibited [9].

Stringent control of autophagy is crucial for Ras-expressing cells as their viability depends on the integrity of the mitochondria structure [10–12]. Autophagy is the mechanism that eliminates the damaged mitochondria and provides the normal ones with substrates for their activity [10,13]. Previously, we have shown that Ras-transformed cells respond to MEK/ERK suppression by activating AMPK-regulated autophagy [14]. However, mTORC1 remains active in these cells despite AMPK activation and the absence of phosphorylated ERK1,2 kinases that contribute to mTORC1 activation [15]. Thus, mechanisms providing a balance between mTORC1, AMPK and autophagy in senescent cells are apparently changed in Ras-transformed cells.

Besides autophagy, mTOR is a key regulator of cellular senescence providing hypertrophic, hyperscretory phenotype of senescent cells by upregulating protein synthesis and formation of SASP as well as down-regulating autophagy [16–18]. Senescence is linked with an increase in mTOR activity, while mTOR inhibitors suppress senescence [19–21]. However, it is less clear how autophagy, mTORC1 and AMPK activity are coordinated in the senescent cells. Although lysosomes co-localize with mTORC1 in perinuclear region of senescent cells [22–24], mTORC1 is constitutively active, regardless of growth factors and nutrients supply [23]. It is also unclear how mTORC1 remains constitutively active and what mechanisms provide “anchoring” the of lysosome-mTORC1 complex nearby nucleus.

Here we studied the effect of mTORC1 suppression on the process of development of AMPK-dependent autophagy arising in response to suppression of MEK/ERK in senescent Ras-expressing cells. mTORC1 suppression by pp242 in senescent Ras–transformed cells results in mitochondria damage and an increase in lysosomal activity. Autophagy ceases in the senescent cells after 24 hours of mTORC1 inhibition by pp242. However, the cells don’t undergo apoptosis due to the formation of non-autophagous LC3 negative vacuoles, which isolate the damaged mitochondria as well as the lysosomes and regulatory proteins like mTOR. Thus, inhibition of mTORC1 in senescent cells is able to activate a rescue mechanism reminiscent of the process of accumulation of autophagic bodies in a special digestive vacuole during yeast starvation. According to our data, senescent cells require MEK/ERK activity to implement this process, as MEK1,2 inhibitor-treated cells fail to complete the rescue mechanism and die.

## RESULTS

### mTORC1 suppression does not increase viability of senescent cells treated by MEK/ERK inhibitor

**Figure 1:**
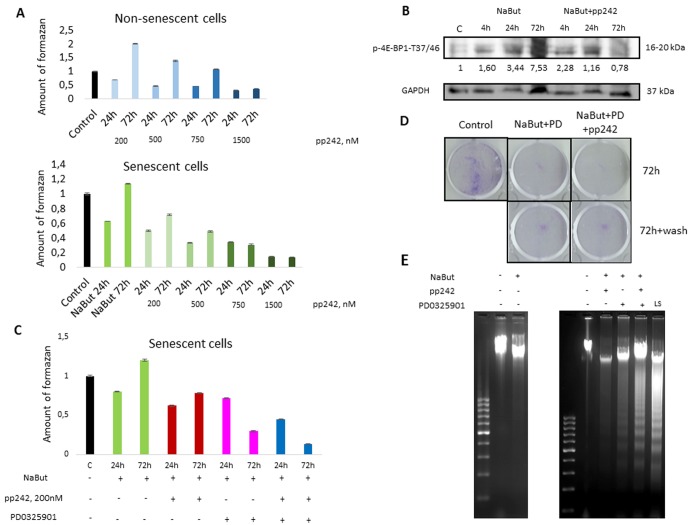
**mTORC1 suppression does not rescue viability of senescent ERas cells exposed to the MEK/ERK inhibitor.** (**A**)Viability of control and senescent ERas cells exposed to mTOR inhibitor pp242 (200 nM, 500 nM, 750 nM, 1500 nM), as assayed by MTT test. (**B**) Suppression of 4E-BP1 phosphorylation by pp242 in senescent ERas cells monitored by Western-blotting. Numbers below represent densitometry of the bands. (**C**) Viability of senescent ERas cells exposed to pp242 (200 nM) and MEK/ERK inhibitor PD0325901 (PD, 1 µM) assayed by MTT test. (**D**) Senescent cells are unable to restore proliferation after MEK/ERK suppression. Cells were exposed to NaBut, PD0325901 and pp242 for 72h then supplemented with a medium without inhibitors for 48 h. Cells were stained with Crystal Violet. (**E**) Senescent ERas cells undergo apoptosis upon mTORC1 and MEK/ERK suppression. DNA fragmentation analysis in 1,5% agarose gel electrophoresis. Serum- starved ERas (LS) were used as positive control for apoptotic DNA fragmentation.

The work was performed using rat embryo fibroblasts, transformed with *E1A+cHa-Ras* oncogenes (ERas cells). Senescence was induced with histone deacetylase inhibitor sodium butyrate (NaBut, 4 mM). Consistent with our previous data, senescent cells are very sensitive to MEK/ERK inhibition, so treatment with specific MEK1,2 kinase inhibitor PD0325901 leads to a significant decrease of cellular viability and apoptotic death [14]. Senescent cells were unable to complete cytoprotective autophagy in response to MEK/ERK suppression. Given that mTORC1 is a negative regulator of autophagy, we used a specific mTOR kinase inhibitor pp242 in 200 nM concentration to suppress mTORC1 activity. The effect of pp242 on cellular viability is concentration-dependent. While cells tolerate the 200 nm concentration, treatment with 1500 nM leads to a significant decrease of cellular viability (Fig. 1A). 200 nM concentration of pp242 decreases phosphorylation of 4E-BP1, a target of mTORC1, after treatment for 72 h (Fig. 1B). It was shown that mTOR inhibitors (pp242, rapamycin, Torin1,2) decelerate senescence [21]. Low concentration of pp242 (200 nM) was used to suppress mTORC1 but only partially decelerate senescence, as deceleration of senescence program leads to proliferation of cells. Our analysis of senescence markers shows that pp242 at 200 nM concentration causes only a partial decrease of senescence markers according to data on Senescence-Associated β-Galactosidase expression and evaluation of the cell size (Suppl. Fig. 1A,B). Senescent cells are characterized with suppression of proliferation. We analyzed cellular regrowth ability after 72 h of pp242 treatment and showed that senescent cells after mTORC1 suppression demonstrate higher ability to proliferate than untreated senescent cells (Suppl. Fig. 1C). Then we questioned whether mTORC1 suppression would rescue viability of senescent cells exposed to MEK/ERK inhibition. However, mTORC1 suppression does not restore cellular viability of senescent cells upon MEK/ERK suppression, as follows from MTT data (Fig. 1C, D, E).

### mTORC1 suppression with 200 nM of pp242 leads to mitochondria damage and increase of lysosomal activity as well as to a transient activation of autophagy

**Figure 2:**
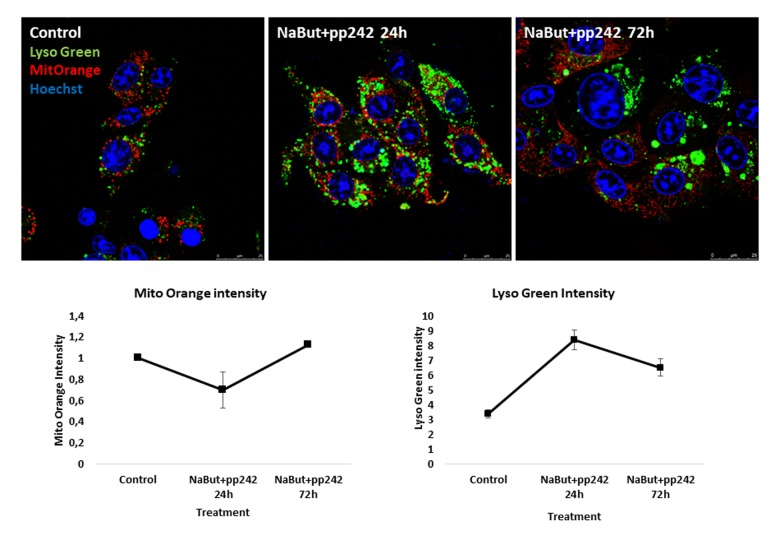
**mTORC1 suppression induces mitochondria damage and an increase of lysosome levels in senescent ERas cells.** Mitochondria are stained with Mitotracker Orange, lysosomes are stained with Lysotracker Green and visualized at proper wavelengths. Nuclei were stained with Hoechst33342. Magnification 40x obj + zoom. Graphs below represent intensities of Mitotracker Orange and Lysotracker Green in control and treated cells, measured using ImageJ software.

Recent reports data have shown that mitochondrial stress affects lysosomal activity [25,26]. In particular, acute mitochondria damage leads to an increase of lysosomal biogenesis [25]. Using in vivo staining with Mitotracker Orange (potential-dependent) and Lysotracker Green, we checked mitochondrial damage and lysosomal activity in senescent cells upon mTORC1 suppression. Data obtained show that mTORC1 suppression leads to mitochondria damage as manifested by a decrease of Mito-Orange signal and in the same time an increase in the lysosome activity (as follows from data acquired using Lysotracker Green) (Fig. 2).

**Figure 3:**
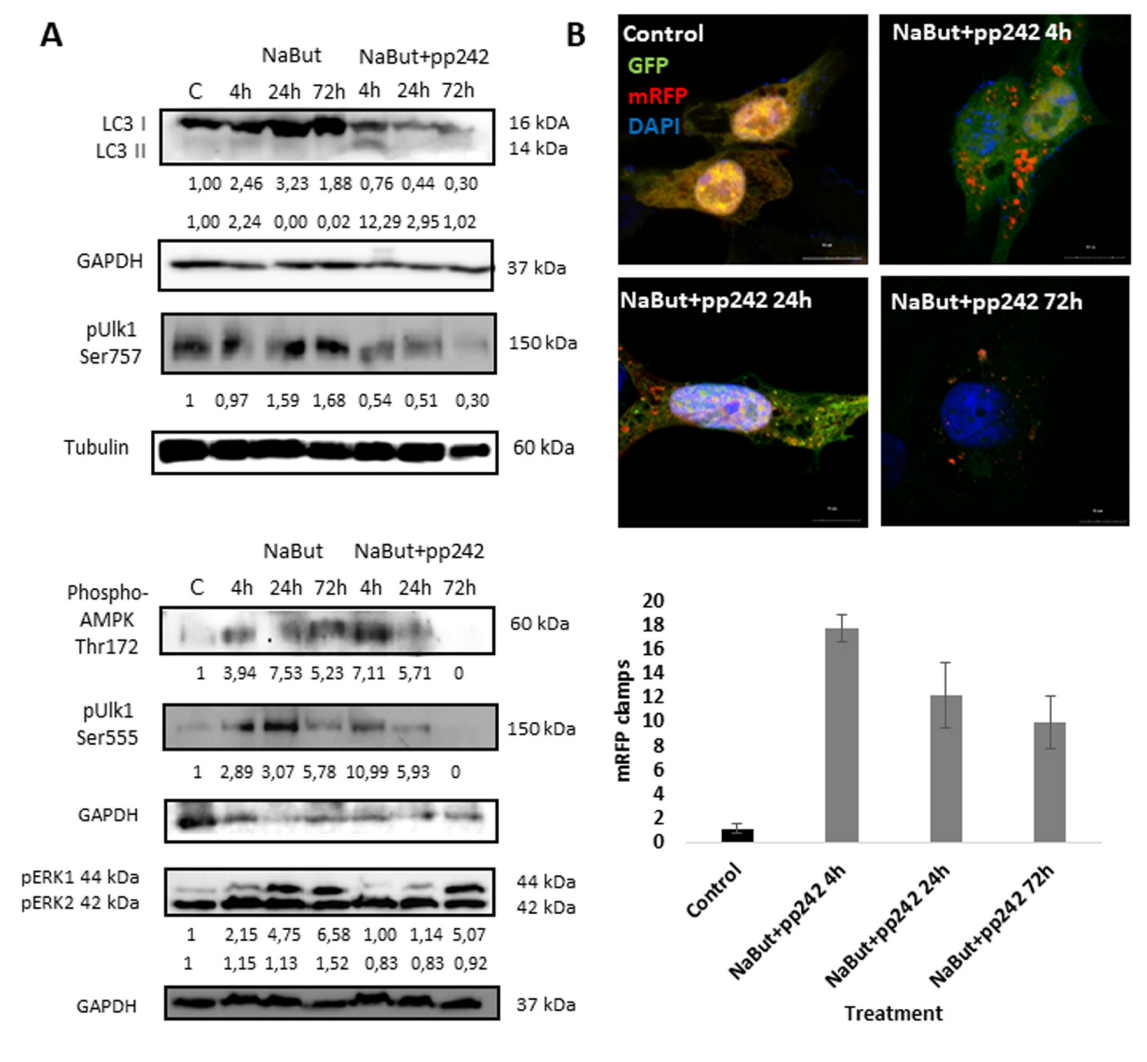
**mTORC1 inhibition induces an autophagy flux, which then terminates 24 h thereafter.** (**A**) Western-blot analysis of autophagy markers and its modulators: LC3 I and II forms, phospho-Ulk1 (Ser757, Ser555), phospho-AMPK (Thr172) as well as ERK1,2 phosphorylation. (**B**) Expression of GFP-mRFP-pft-LC3 vector in senescent cells exposed to pp242 for 4, 24, 72 h visualizing formation of autophagosomes and fusion of autophagosomes with lysosomes. The nuclei stained with DAPI. Histogram presents the average number mRFP foci (autophagosomes fused with lysosomes) per cell after 4, 24, 72 h of mTORC1 inhibition.

Mitochondria damage results in induction of autophagy, as demonstrated by an increase of LC3 I to LC3 II conversion after treatment with pp242 for 4 h (Fig. 3A). To analyze the completeness of autophagy induced by mTORC1 inhibition, we transfected cells with a plasmid that encodes LC3 fused with GFP and mRFP (Addgene #21074). When autophagosomes fuse with lysosomes, GFP-LC3 fluorescence vanishes due to acidification [27], thus allowing to distinguish autophagosomes from autophagolysosomes and to estimate the completeness of autophagic process. In control cells, both GFP and RFP signals distribute diffusely in the cytoplasm (Fig. 3B). Given that GFP signal disappears, whereas the clamps of mRFP arise after mTORC1 suppression with pp242 for 4 h, this evidence that the autophagosomes are formed and fuse with the lysosomes. However, later (24 h - 72 h) the number of mRFP clamps decreases, implying that formation of autophagosomes is attenuated (Fig. 3B).

### ERas cells treated with mTOR inhibitor cease autophagy and eliminate damaged mitochondria through an alternative pathway

**Figure 4:**
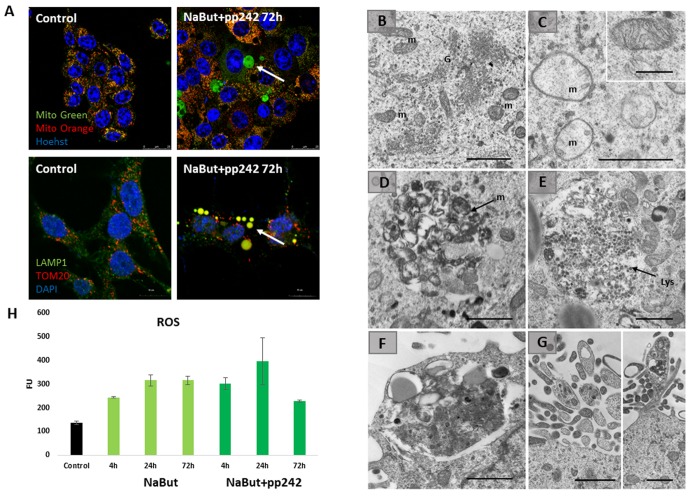
**Senescent cells segregate damaged mitochondria into LC3-negative vacuoles after mTORC1 suppression.** (**A**) IF images showing segregation of damaged mitochondria in the specific LC3-negative vacuoles in senescent cells exposed to mTOR inhibitor for 72 h. Upper panel: *in vivo* staining with Mitotracker Green and Mitotracker Orange visualized with the Leica TSC Microscope, 40x obj + zoom. Nuclei stained with Hoechst 33342. White arrow points the damaged mitochondria (Mitotracker Green only) inside of the vacuoles. Bottom panel: IF pictures after staining with antibodies against TOM20 and LAMP1 taken on the Olympus Fluoview 3000, 60x obj. Nuclei stained with DAPI. Arrow points the mitochondria colocalized with the lysosomes. (**B**) TEM image of senescent ERas cell with active mTORC1 (72 h) exhibiting the non-damaged mitochondria (m) and the enriched Golgi complex (G). (**C**) TEM image of senescent ERas cell treated with mTOR inhibitor pp242 for 72 h exhibiting the damaged mitochondria (m). Inset presents normal non-damaged mitochondria of untreated, non-senescent ERas cell. (**D**) Accumulation of membranous structures (mitochondria remnants, m) in the vacuole of senescent cell upon mTORC1 suppression. (**E**) Accumulation of lysosomes (Lys) in the vacuole of senescent cell upon mTOR suppression. (**F**) The vacuole containing lysosomes and membrane structure close to the plasma membrane of senescent ERas cell treated with mTOR inhibitor. (**G**) Membrane and electron-dense structures excreted from the cell. Scale bars in TEM images: 1 µm; inset (B) – 0,5 µm. (**H**) The level of reactive oxygen species (ROS) in senescent cells after 72 h of mTOR suppression measured using DCF-DA at proper wavelength.

Given that upon 24 h of mTORC1 suppression autophagy is disrupted, the cells fail to degrade the damaged mitochondria. To find out how the damaged mitochondria are degraded, we performed Transmission Electron Microscopy (TEM) as well as in vivo staining with Mito-tracker Green (binds to all mitochondria) and Mitotracker Orange (binds only non-damaged mitochondria that retain their membrane potential). For IF staining, antibodies against lysosomal protein (LAMP1) and transmembrane protein on the outer membranes of mitochondria (TOM20) were used. TEM data showed abundance of damaged mitochondria with severe disorders of cristae in senescent cells with suppressed mTORC1 (Fig. 4C, D, F). However, after 72 h the vacuoles containing numerous membrane structures can be seen, pointing that the damaged mitochondria concentrate in the vacuoles (Fig. 4C, D, F). As shown by Mitotracker Green staining data as well from data acquired in the combined staining with Mitotracker Orange and Lysotracker Green, these mitochondria are in fact damaged. This follows from the results that no Mitotracker Orange signal is revealed in the vacuoles, where LysoGreen clamps reside. Onwards, TEM images show that cells exhibit intense exfoliations at their edges, where some membrane structures, probably, mitochondria remnants can be seen (Fig. 4E, F). Nevertheless, in the cytoplasm outside of the clamps normal mitochondria are visualized, as Mitotracker Orange intensity, that was decreased after 24 h of treatment, returns almost to the control values after 72 h, implying the recovery of functioning mitochondria located outside of the vacuoles. Senescent cells exhibit an increase of the oxygen species (ROS) levels. Mitochondria damaged upon mTORC1 suppression are a source of ROS. However, after 72 h of mTORC1 inhibition the ROS levels become lower as compared with senescent cells retaining active mTORC1, thereby indicating that there is a rescue mechanism that counters accumulation of ROS through isolation of the damaged mitochondria (Fig. 4H).

**Figure 5:**
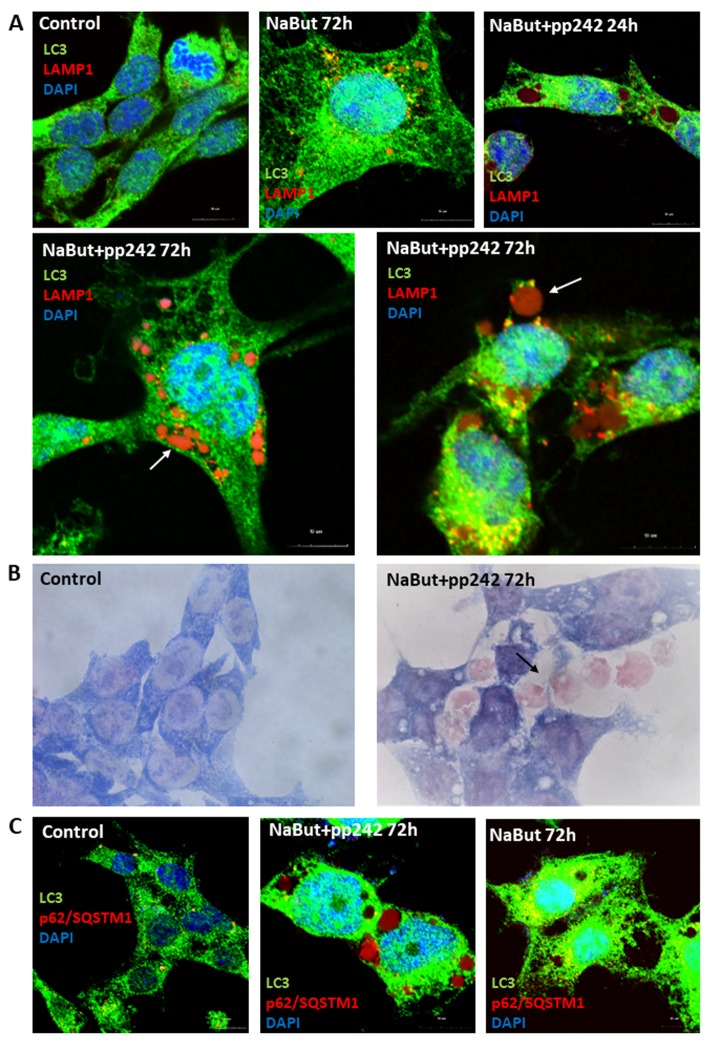
**Senescent pp242-treated cells with disrupted autophagy form the LC3-negative cavities where lysosomes and p62/SQSTM1 accumulate.** (**A**) IF analysis of LC3 and lysosome (LAMP1) localization in senescent cells and mTORC1- suppressed senescent cells. Arrows show LAMP1 clamps in the LC3-negative vacuole (left image) and outside of the cell (right image). Nuclei stained with DAPI. (**B**) Morphology of senescent cells after 72 h of mTORC1 inhibition. Arrow shows acidic clamps secreted from the cells. (**C**) P62/SQSTM1 accumulates in vacuoles of senescent cells exposed to pp242. Nuclei stained with DAPI.

**Figure 6:**
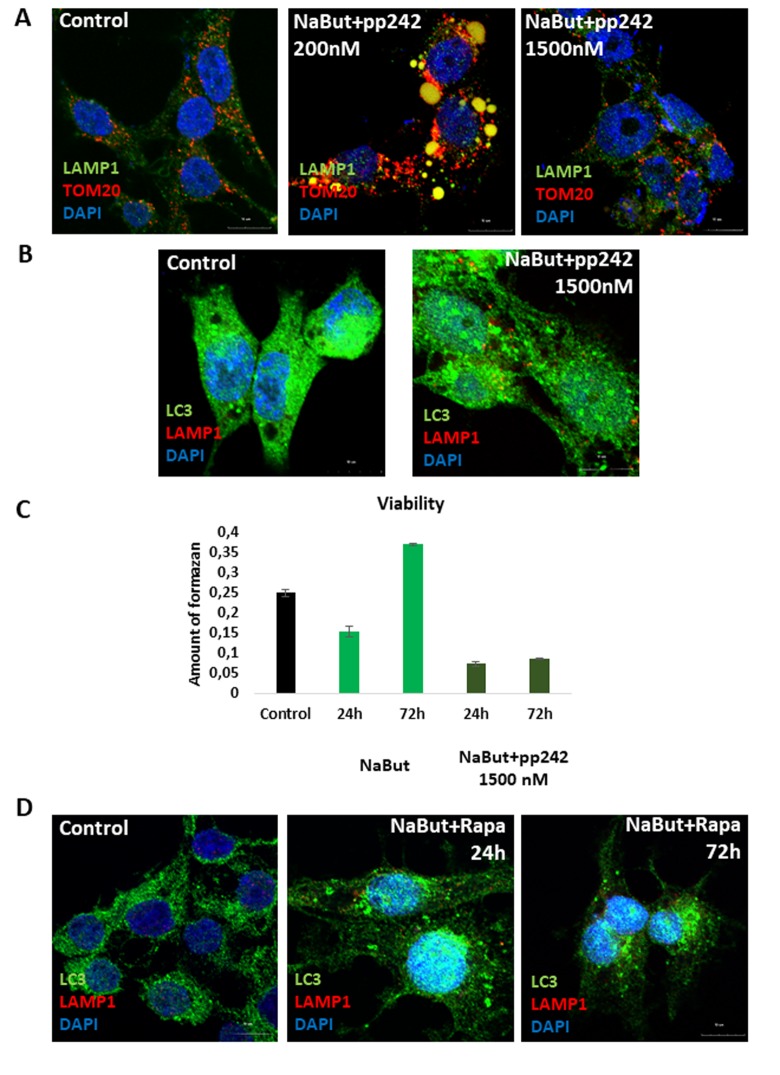
**Senescent cells exposed to high concentration of mTOR inhibitor pp242 (1500 nM) fail to rescue themselves by forming the LC3-negative vacuoles and undergo cell death.** (**A**) IF pictures after staining with antibodies against TOM20 and LAMP1. The mitochondria and lysosomes are segregated in the vacuoles of cells exposed to 200 nM pp242, but not to 1500 nM pp242. Nuclei stained with DAPI. (**B**) IF with antibodies against LAMP1 and LC3. (**C**) Viability of senescent ERas cells treated with 1500 nM pp242 and assayed by MTT test. (**D**) IF with antibodies against LAMP1 and LC3 showing the absence of the mitochondria and lysosomes-containing vacuoles after mTORC1 suppression with 200 nM of rapamycin.

IF analysis with antibodies against a lysosome marker LAMP1 showed that these vacuoles contains LAMP1 positive clamps, implying that lysosomes also accumulate therein (Fig. 5A). The results of immunofluorescence are confirmed by data on morphological Giemza staining that show acidic inclusions within the vacuoles of the cells as well as outside of the cells (Fig. 4B). LAMP1 immunofluorescence shows that after mTORC1 suppression with pp242 for 72 h the LAMP1 and TOM20 fully colocalizes in the clamps that is a direct evidence for aggregation of mitochondria and lysosomes within the large vacuoles (Fig. 4A). Transmission electron microscopy also revealed big vacuoles filled with lysosome-like cargo, thereby confirming data acquired with Lyso-tracker Green and LAMP1 staining (Fig. 4E). However, no LC3 signal is observed inside of the vacuoles together with LAMP1. Moreover, the senescent ERas cells transfected with LC3-GFP/RFP plasmid (ERas-LC3 cells) and treated with pp242 demonstrate neither GFP-LC3, nor mRFP-LC3 aggregations (Fig. 3B). This proves that the vacuoles are devoid of autophagosomes and autophagolysosomes. Analysis of p62/SQSTM1 shows its presence is discovered in the vacuoles, but not in co-localization with the LC3 (Fig. 5C). In case of canonical autophagy, when autophagic process is successfully finished, p62/SQSTM1 degrades together with the autophagolysosomal cargo. In senescent cells with suppressed mTORC1 activity, autophagy is interrupted, therefore, p62/SQSTM1 does not degrade and redistributes to the vacuoles. Thus, mTORC1 suppression with low doses of pp242 induces mitochondrial damage in senescent cells, but the cells get rescued by a special mechanism when damaged mitochondria are placed in the vacuoles together with the lysosomes. However, upon increased concentration of pp242 (1500 nM) the cells fail to implement this mechanism and die by apoptosis (Fig. 6A, B, C). Data obtained are consistent with the fact that viability of Ras-expressing cells is dependent on mitochondria, and processes observed in cells treated with low concentrations of pp242 result in elimination of damaged mitochondria. We’ve applied mTORC1 inhibitor rapamycin that doesn’t cause mitochondria damage, according to our previous works. Senescent cells treated with rapamycin don’t reveal vacuole formation as cells treated with 200 nM pp242 (Fig. 6D). The absence of mitochondria damage in cells treated with rapamycin might be explaining the differences in responses to pp242 and rapamycin.

**Figure 7:**
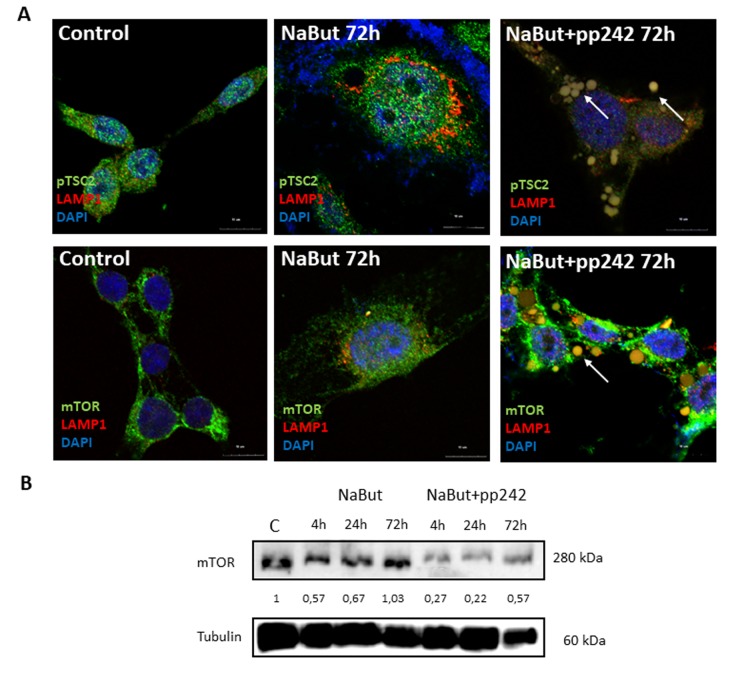
**mTOR inhibition with pp242 uncouples mTOR from the lysosomes.** (**A**) IF images showing pTSC2 (upper panel), mTOR (bottom panel) and lysosome (LAMP1) localization in senescent cells treated with pp242. Arrows show segregation of mTOR and pTSC2 (upper) and mTOR together with lysosomes (lower) in vacuoles and outside of cells. Nuclei stained with DAPI. (**B**) Western-blot analysis of mTOR levels in senescent cells after 72 h of pp242 treatment. The numbers represent densitometry of the bands.

Several groups reported that mTORC1 is localized on the surface of lysosomes [7]. IF analysis of senescent cells shows that after pp242 treatment for 72 h a small part of mTOR is detected in the vacuoles together with the lysosomes (Fig. 7A). Besides, mTORC1-lysosome aggregates can be seen outside the cells, and the total amount of mTOR decreases, according to Western-blot data (Fig. 7B). However, the most part of mTOR remains around the nucleus outside of the LAMP1-positive vacuoles, and distribution of the mTOR signals are clearly uncoupled from the LAMP1 signals (Fig. 7A). Therefore, mTORC1-lysosome interaction is weakened. Main negative regulator of mTORC1 – TSC2 protein, was also found in the vacuoles. Thus, the formation of the large vacuoles, where damaged mitochondria and lysosomes are segregated and then removed from the cell, can be a novel rescue mechanism. According to MTT data, this mechanism provides nearby 70% survival after 120h of treatment.

### Senescent cells require active MEK/ERK to implement non-autophagous rescue pathway

**Figure 8:**
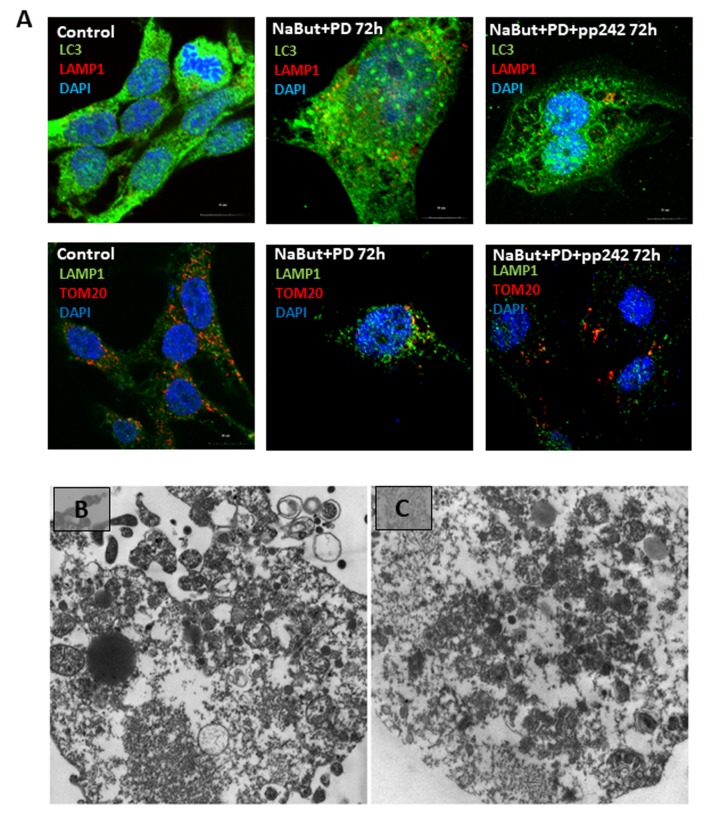
**Senescent cells require active MEK/ERK pathway to implement degradation of the damaged mitochondria through their segregation into the LC3-negative vacuoles.** (**A**) Cells exposed to MEK/ERK inhibitor do not segregate of lysosomes in the LC3-negative vacuoles (upper panel). IF images after staining with antibodies against LC3 and LAMP1) or mitochondria (bottom panel), IF images after staining with antibodies against TOM20 and LAMP1) of the LC3-negative vacuoles. (**B**) TEM of a senescent cell after MEK/ERK suppression undergoing complete destruction of the cytoplasm without segregation and elimination of the damaged mitochondria. (**C**) TEM image of senescent cells exposed to pp242 + PD0325901 showing destruction of the cytoplasm and dispersed damaged mitochondria. Scale bars: 1 µm.

Ras-transformed cells exhibit constitutive activity of downstream Raf/MEK/ERK pathway. According to the Western-blot data, MEK/ERK activity retains on a high level in senescent cells regardless of mTORC1 activity (Fig. 3A). IF data show that senescent cells with suppressed MEK/ERK (NaBut + PD0325901) do not reveal accumulation of mitochondria and lysosomes in the content of the large vacuoles (Fig. 8A). Suppression of mTORC1 in senescent cells with inhibited MEK/ERK activity does not lead neither to restoration of autophagy nor to the large vacuole formation. This result is supported by TEM that shows the damaged mitochondria are distributed within the cell, but no vacuoles do appear (Fig. 8B, C). As follows from the MTT, 120 h treatment triggers almost 90% of cells to undergo apoptosis, thereby pointing out that senescent cells are more sensitive to MEK/ERK inhibitor than to mTOR inhibitor. One may conclude that active MEK/ERK pathway in ERas cells is indispensable to implement the mechanism of the LC3-lacking vacuole formation.

## DISCUSSION

Autophagy is an evolutionary conservative mechanism that maintains cellular viability by regulating the quantity and quality of organelles, including mitochondria, and macromolecules [28]. Recently it was found that negative regulator of autophagy mTORC1 signaling also controls mitochondrial dynamics to govern the cell fate decisions, so that mTORC1 inhibition by Torin1 and rapamycin treatment leads to mitochondrial branching and hyperfusion through 4E-BP1-dependent suppression of MTFP1 mRNA translation [29–31]. For Ras-expressing tumor cells, unlike other tumor cell lines, maintenance of the mitochondrial integrity is crucial as they were shown to rely on mitochondria as energy source rather than glycolysis [10–13]. On the other hand, mitochondrial activity is also implicated in senescence, as depletion of mitochondria was shown to slow down Ras-induced senescence in murine cell lines [32]. Senescence is linked with mitochondria dysfunction and correlates with accumulation of ROS levels and decrease of energy level that stimulate AMPK activity [33] which upregulates autophagy to withstand mitochondrial dysfunction. Thus, viability of senescent cells is a consequence of a balance of AMPK and mTORC1 activities as well as mitochondrial integrity.

Senescent cells exhibit active mTORC1 and at the same time elevated AMPK activity. So, the question arises, how would mTORC1 inhibition affect AMPK-regulated autophagy. Our results show that mTORC1 suppression even with low concentration of pp242 (200nM) in senescent cells leads to mitochondria damage. Rapamycin, as our previous work shows, does not cause mitochondria damage, that might be explain the differences in responses to pp242 and rapamycin. Upon 200 nM pp242 mTORC1-dependent autophagy is induced, however, it stops after 24 h of mTORC1 inhibition. Thus, decrease of mTORC1 activity might not guarantee autophagy completion. To maintain viability, cells induce a mechanism for isolating the damaged organelles into the large enclosed and LC3 lacking vacuoles. These vacuoles resemble vacuoles that are observed in the yeasts upon starvation [34]. The case of LC3-independent autophagic degradation process was also detected on hepatocyte model, showing macroautophagic sequestration to be dependent on GABARAP rather than LC3 [35]. According to our data, the large vacuoles only partially release the contents outside the cell, whereas the remaining proteins and organelles might probably be digested by lysosomal enzymes directly within the vacuoles. So, the vacuoles might represent a novel specific mechanism of degradation of the damaged organelles provided that autophagy is compromised. But upon MEK/ERK suppression neither autophagy nor abovementioned mechanism is present and no rescue of viability is observed upon either pp242 or rapamycin. Thus, the conclusion is that mTORC1 suppression doesn’t rescue viability of senescent cells exposed to MEK/ERK inhibitor.

Another property of senescent cells is specific spatial organization of cytoplasm with lysosomes localized around nucleus, providing separation of lysosomes and autophagosomes and low lysosome-autophagosome fusion level to maintain hypertrophy. An important manifestation of this property is providing a balance between AMPK and mTORC1. Given that AMPK is a negative regulator for mTORC1, it is obvious to question how mTORC1 can remain active in the presence of active AMPK. There can be a feedback loop down from mTORC1 to AMPK. Nevertheless, a report by Mukhopadhyay et al shows that Phospholipase D (PLD) can suppress AMPK by acting downstream of mTORC1, thereby implying the existence of such feedback loop [36]. But of most special interest is the role of the lysosomes in maintaining activity of mTORC1 or AMPK. It was shown and discussed that a switch between mTORC1 and AMPK on the lysosomal surface is mediated by the v-ATPase-Ragulator complex, which is capable of activating either mTORC1 or AMPK [7]. However, the “decision” to switch and how this switch is modulated in senescent cells remains to be studied in more details. Nevertheless, the analysis of lysosomes and mTORC1 positioning in conditions of starvation gives some insight on how this regulation takes place. The maintenance of active mTORC1 on lysosomes at the periphery of non-senescent cells is dependent on kinesins KIF1Bβ and KIF2 and small GTPase ARL8B [37]. Nutrient starvation as well as knockdown of the above proteins resulted in relocalization of lysosomes close to the nucleus and the microtubule-organization center as well as in reduction of mTORC1 activity [37]. Thus, one may suppose that in senescent cells kinesin and ARL8B activity is reduced to provide lysosome clustering near the nucleus. However, in senescent cells mTORC1 remains active in the perinuclear region regardless of nutrients and growth factors deficiency. The main negative regulator of mTORC1 activity dimeric complex TSC1,2 is also translocated to the lysosomal surface to down-regulate mTORC1. But in senescent cells mTORC1 is active despite TSC2 co-localization. In senescent cells, factors that alter lysosomal positioning do not depend on the changes in nutrient availability; however, they may depend on mTORC1 activity. We showed that a part of lysosomes in pp242-treated cells is found in the vesicles clearly separated from mTOR. Therefore, mTORC1-lysosomal positioning in senescent cells is only partly regulated by mTOR itself. Of interest, according to Korolchuk et al, mTORC2 is not associated with lysosomes, unlike mTORC1 [9]. So, these data give new insights in mTORC1/mTORC2-AMPK-lysosome network in senescent cells. Nevertheless, the important result is that spatial organization of senescent cells does not allow to complete autophagy even in case of mitochondria damage upon mTORC1 suppression. As we obtained in previous work, lysosomes in senescent cells don’t change their localization upon MEK/ERK suppression [14]. Here we show that the vulnerability of senescent cells is rather spatial organization of cytoplasm than high activity of mTORC1. So, spatial organization of cytoplasm of senescent cells can be a factor that increases sensitivity to MEK/ERK inhibitor.

We show here that cells with suppressed MEK/ERK fail to complete the rescue pathway of isolating mitochondria and lysosomes into the large vacuoles. ERK2 kinase is involved into regulation of lysosomal biogenesis [38,39]. Authors have shown that inhibition of ERK1,2 phosphorylation allows nuclear translocation of TFEB and up-regulation of lysosome biogenesis. However, in ERas cells upon MEK/ERK suppression the lysosome signal is weakened and no LAMP1-positive inclusions are observed, pointing that in these cells lysosome regulation may differ. Senescent cells also differ in case of lysosomal regulation, as they exhibit increase of lysosomal mass together with high constitutive activity of mTORC1, which was shown to suppress nuclear translocation of TFEB in non-senescent cells. So, the aspects of regulation of lysosomal activity in senescent cells are still to investigate, but understanding them gives new understanding of metabolic features of senescent cells which can be used for selective killing Ras-expressing cells.

## MATERIALS AND METHODS

### Cells

Rat embryonic fibroblasts were co-transformed with *E1A* region of Ad5 DNA and human *cHa-Ras* oncogene (Q12 and G61 mutated). Cells were cultured in DMEM with 10% FBS (HyClone) in 5% CO2 at 37 ^°^C. Histone deacetylase inhibitor sodium butyrate (NaBut, 4 mM, Sigma) was used to induce senescence. For mTOR suppression, specific kinase inhibitor pp242 was used in 200nM concentration. PD0325901 (PD, 1 µM, Sigma) was used to inhibit MEK1,2 activity and ERK1,2 phosphorylation.

### Cell viability

For MTT test cells were seeded at 12-well plate at initial density 2x10^4^ cells per well and treated with inhibitors for indicated time. Then medium was changed for 0,5 mg/ml MTT solution for 1h at 37C. Then formazan produced was dissolved in DMSO and measured at 572 nm wavelength. For regrowth analysis, cells were seeded on 12-well plates, treated with inhibitors for 72h, then supplemented with fresh medium without inhibitors. After 48h of cultivation cells were stained with Crystal Violet.

### Cellular transfection

Cells were transfected with pft-LC3 plasmid (Addgene #21074) using Lipofectamine 2000 as prescribed by manufacturer (Thermo Fischer, 11668027).

### Nucleosomal DNA fragmentation assay

Cells were seeded at 100mM dishes and treated with inhibitors, then lysed in TES buffer containing 10 mM Tris-HCl (pH 8,0), 0,1M EDTA (pH 8,0) and 0,5% SDS, supplemented with RNAse A and NaCl at 37C, then left overnight with Proteinase K and SDS. Next day DNA was deproteinized by a mixture of water-saturated phenol pH 8.0/chloroform/izoamyl alcohol. DNA was reprecipitated, washed with 70% of ethanol, dried and dissolved in 1 mM TE buffer pH 8.0. DNA was subjected to electrophoresis in 2% agarose gel (Sigma). DNA bands were stained with ethidium bromide. DNA isolated from ERas cells starved for 24 h in 0.5% FBS undergoing apoptosis [40] was used as a positive control.

### Immunofluorescence

Cells were seeded at coverslips and treated for indicated time, that fixed, permeabilized with 0,2% TRITON X100, blocked in 3% BSA in TBS-T, and incubated in primary antibodies overnight at +4°C following the incubation with secondary antibodies for how long and what temperature. Coverslips were mounted with DAPI-containing ProLong Gold mounting medium (Molecular Probes, P36931). Cells were visualized using Olympus Fluoview 3000 microscope (Olympus).

### Western blotting

Cells were lysed in RIPA buffer (1% IGEPAL, 0,5% Sodium deoxycholate; 0,1% SDS; 50 mM TRIS-HCl pH 8,0; 150 mM sodium chloride; 5 mM EDTA pH 8,0; 60 mM sodium fluoride) + the Roche Protease inhibitor cocktail. 50 µg of protein were separated by SDS-PAGE electrophoresis in 12,5%, 12% or 10% gels and transferred to PVDF membranes (0,2 and 0,45 µM, EMD Millipore). Membranes were blocked with 5% fat-free milk (Sigma) for 1 h following the incubation with primary antibodies in TBS solution containing 3% BSA and 0,1% Tween-20 overnight at 4°C. The membranes were washed and incubated with secondary antibodies conjugated with horseradish peroxidase. Blots were developed by enhanced chemiluminescence (ECL, ThermoFisher Scientific). Densitometry was performed using GelPro3 Software (Media Cybernetics).

### Lysosome detection by Lysotracker Green

For lysosome detection, Lysotracker Green was used as prescribed by manufacturer (Thermo Fischer Scientific, L7526). Nuclei were stained with Hoechst33342 (Thermo Fischer Scientific, 62249). Images were obtained using Leica TSC5 microscope (Leica Microsystems) and analyzed using ImageJ software.

### Mitotracker Orange and Mitotracker Green staining for detection of mitochondrial integrity

Cells were seeded on coverslips and treated with the inhibitors for indicated time, then stained with Mitotracker Orange and Mitotracker Green (Invitrogen, M7510 and M7514) as described by manufacturer. The intensity of fluorescence was visualized using Leica TCS SP5 microscope (Leica Microsystems) at following wavelengths: Ex/Em=577/599 nm for Mitotracker Orange, 490/516 nm for Mitotracker Green. Images were analyzed using ImageJ software.

### ROS levels

Cell were treated with inhibitors for the indicated time points and then incubated with 10 μM 2′-7′-dichlorodihydrofluorescene diacetate (DCFDA, Invitrogen D399) for 30 min. DCF-DA fluorescence was measured using FLUOstar Omega (BMG Labtech) microplate reader and normalized to cell number.

### Senescence-associated β-galactosidase detection

Sa-β-Gal expression was analyzed as previously described [41]. Images were acquired using LSM5 Pascal microscope (Carl Zeiss Microscopy).

### Cell size

Cell were seeded on 60mm dishes and treated with inhibitors for 72h, then collected and forward scattering was measured using Odam (Brucker, France) flow cytometer.

### Transmission electron microscopy

Cells adhered to the coverslips were mechanically detached and fixed in 2.5% glutaraldehyde in 0.1M cacodylate buffer, pH 7.2-7.4, for 1 h at 4°C, postfixed in 1% aqueous OsO4 for 1 h, dehydrated, and embedded in Epon and Araldit, and then sectioned with a diamond knife on a LKB-ultratome (Sweden). Ultrathin sections were collected on fine mesh copper grids, and stained with uranyl acetate and lead citrate for examination with a Zeiss Libra 120 electron microscope (Carl Zeiss, Germany) at an accelerating voltage of 80 kV.

### Statistical analysis

Confocal images were analyzed using ImageJ software; densitometry of Western Blotting was performed using free distributed GelPro Analyzer software. Data are presented as means ± S.E.M of three independent experiments.

### Antibodies

The following primary antibodies were used: pan-LC3 (MBL, #PM036); LAMP1 (Santa-Cruz, sc-17768); phospho-Ulk1 Ser757 (Cell Signaling, #6888S); phospho-Ulk1 Ser555 (Cell Signalling, #5869); Tom20 (Santa-Cruz, sc-17764); phospho-AMPK T172 (Cell Signaling, #2535S); phospho-4E-BP1 Thr37/46 (Cell Signaling, #2855S); phospho-42/44 MAPK Thr202/Tyr204 (Cell Signaling, #4377S); mTOR (Cell Signaling, #2983S); pTSC2 (Cell Signalling, #3617), GAPDH (Cell signaling, #2118), Tubulin (Sigma, T5168). The following secondary antibodies were used: Goat-anti-Rabbit IgG (H+L) Alexa Flour 488 (Invitrogen, A11088); Rabbit-anti-Mouse IgG (H+L) Alexa Fluor 568 (Invitrogen, A11031); Goat-anti-Rabbit IgG HRP Conjugated (Sigma-Aldrich, A0545); Rabbit-anti-Mouse IgG HRP Conjugated (Sigma-Aldrich, A9044).

## Supplementary Material

Supplementary Figure

## References

[1] López-Otín C, Blasco MA, Partridge L, Serrano M, Kroemer G. The hallmarks of aging. Cell. 2013; 153:1194–217. 10.1016/j.cell.2013.05.039 23746838 PMC3836174

[2] Laplante M, Sabatini DM. mTOR signaling in growth control and disease. Cell. 2012; 149:274–93. 10.1016/j.cell.2012.03.017 22500797 PMC3331679

[3] Kim J, Kundu M, Viollet B, Guan KL. AMPK and mTOR regulate autophagy through direct phosphorylation of Ulk1. Nat Cell Biol. 2011; 13:132–41. 10.1038/ncb2152 21258367 PMC3987946

[4] Hosokawa N, Hara T, Kaizuka T, Kishi C, Takamura A, Miura Y, Iemura S, Natsume T, Takehana K, Yamada N, Guan JL, Oshiro N, Mizushima N. Nutrient-dependent mTORC1 association with the ULK1-Atg13-FIP200 complex required for autophagy. Nat Cell Biol. 2009; 20:1981–91. 10.1091/mbc.E08-12-1248 19211835 PMC2663915

[5] Lee JW, Park S, Takahashi Y, Wang HG. The association of AMPK with ULK1 regulates autophagy. PLoS One. 2010; 5:e15394. 10.1371/journal.pone.0015394 21072212 PMC2972217

[6] Egan DF, Shackelford DB, Mihaylova MM, Gelino S, Kohnz RA, Mair W, Vasquez DS, Joshi A, Gwinn DM, Taylor R, Asara JM, Fitzpatrick J, Dillin A, et al. Phosphorylation of ULK1 (hATG1) by AMP-activated protein kinase connects energy sensing to mitophagy. Science. 2011; 331:456–61. 10.1126/science.1196371 21205641 PMC3030664

[7] Carroll B, Dunlop EA. The lysosome: a crucial hub for AMPK and mTORC1 signalling. Biochem J. 2017; 474:1453–66. 10.1042/BCJ20160780 28408430

[8] Zhang CS, Jiang B, Li M, Zhu M, Peng Y, Zhang YL, Wu YQ, Li TY, Liang Y, Lu Z, Lian G, Liu Q, Guo H, et al. The lysosomal v-ATPase-Ragulator complex is a common activator for AMPK and mTORC1, acting as a switch between catabolism and anabolism. Cell Metab. 2014; 20:526–40. 10.1016/j.cmet.2014.06.014 25002183

[9] Korolchuk VI, Saiki S, Lichtenberg M, Siddiqi FH, Roberts EA, Imarisio S, Jahreiss L, Sarkar S, Futter M, Menzies FM, O’Kane CJ, Deretic V, Rubinsztein DC. Lysosomal positioning coordinates cellular nutrient responses. Nat Cell Biol. 2011; 13:453–60. 10.1038/ncb2204 21394080 PMC3071334

[10] Guo JY, Chen HY, Mathew R, Fan J, Strohecker AM, Karsli-Uzunbas G, Kamphorst JJ, Chen G, Lemons JM, Karantza V, Coller HA, Dipaola RS, Gelinas C, et al. Activated Ras requires autophagy to maintain oxidative metabolism and tumorigenesis. Genes Dev. 2011; 25:460–70. 10.1101/gad.2016311 21317241 PMC3049287

[11] Guo JY, White E. Autophagy is required for mitochondrial function, lipid metabolism, growth, and fate of KRAS(G12D)-driven lung tumors. Autophagy. 2013; 9:1636–38. 10.4161/auto.26123 23959381 PMC5424446

[12] Schmukler E, Kloog Y, Pinkas-Kramarski R. Ras and autophagy in cancer development and therapy. Oncotarget. 2014; 5:577–86. 10.18632/oncotarget.1775 24583697 PMC3996671

[13] Guo JY, Teng X, Laddha SV, Ma S, Van Nostrand SC, Yang Y, Khor S, Chan CS, Rabinowitz JD, White E. Autophagy provides metabolic substrates to maintain energy charge and nucleotide pools in Ras-driven lung cancer cells. Genes Dev. 2016; 30:1704–17. 10.1101/gad.283416.116 27516533 PMC5002976

[14] Kochetkova EY, Blinova GI, Bystrova OA, Martynova MG, Pospelov VA, Pospelova TV. Targeted elimination of senescent Ras-transformed cells by suppression of MEK/ERK pathway. Aging (Albany NY). 2017; 9:2352–75. 10.18632/aging.101325 29140794 PMC5723691

[15] Ma L, Chen Z, Erdjument-Bromage H, Tempst P, Pandolfi PP. Phosphorylation and functional inactivation of TSC2 by Erk implications for tuberous sclerosis and cancer pathogenesis. Cell. 2005; 121:179–93. 10.1016/j.cell.2005.02.031 15851026

[16] Blagosklonny MV. Cell cycle arrest is not senescence. Aging (Albany NY). 2011; 3:94–101. 10.18632/aging.100281 21297220 PMC3082019

[17] Blagosklonny MV. Molecular damage in cancer: an argument for mTOR-driven aging. Aging (Albany NY). 2011; 3:1130–41. 10.18632/aging.100422 22246147 PMC3273893

[18] Blagosklonny MV. Cell cycle arrest is not yet senescence, which is not just cell cycle arrest: terminology for TOR-driven aging. Aging (Albany NY). 2012; 4:159–65. 10.18632/aging.100443 22394614 PMC3348476

[19] Demidenko ZN, Zubova SG, Bukreeva EI, Pospelov VA, Pospelova TV, Blagosklonny MV.Rapamycin decelerates cellular senescence. Cell Cycle. 2009; 8:1888–95. 10.4161/cc.8.12.8606 19471117

[20] Leontieva OV, Demidenko ZN, Blagosklonny MV. Dual mTORC1/C2 inhibitors suppress cellular geroconversion (a senescence program). Oncotarget. 2015; 6:23238–48. 10.18632/oncotarget.4836 26177051 PMC4695114

[21] Leontieva OV, Blagosklonny MV. Gerosuppression by pan-mTOR inhibitors. Aging (Albany NY). 2016; 8:3535–51. 10.18632/aging.101155 28077803 PMC5270685

[22] Narita M, Young AR, Arakawa S, Samarajiwa SA, Nakashima T, Yoshida S, Hong S, Berry LS, Reichelt S, Ferreira M, Tavaré S, Inoki K, Shimizu S, Narita M. Spatial coupling of mTOR and autophagy augments secretory phenotypes. Science. 2011; 332:966–70. 10.1126/science.1205407 21512002 PMC3426290

[23] Young AR, Narita M, Narita M. Spatio-temporal association between mTOR and autophagy during cellular senescence. Autophagy. 2011; 7:1387–88. 10.4161/auto.7.11.17348 21799306

[24] Carroll B, Nelson G, Rabanal-Ruiz Y, Kucheryavenko O, Dunhill-Turner NA, Chesterman CC, Zahari Q, Zhang T, Conduit SE, Mitchell CA, Maddocks OD, Lovat P, von Zglinicki T, Korolchuk VI. Persistent mTORC1 signaling in cell senescence results from defects in amino acid and growth factor sensing. J Cell Biol. 2017; 216:1949–57. 10.1083/jcb.201610113 28566325 PMC5496614

[25] Fernández-Mosquera L, Diogo CV, Yambire KF, Santos GL, Luna Sánchez M, Bénit P, Rustin P, Lopez LC, Milosevic I, Raimundo N. Acute and chronic mitochondrial respiratory chain deficiency differentially regulate lysosomal biogenesis. Sci Rep. 2017; 7:45076. 10.1038/srep45076 28345620 PMC5366864

[26] Demers-Lamarche J, Guillebaud G, Tlili M, Todkar K, Bélanger N, Grondin M, Nguyen AP, Michel J, Germain M. Loss of Mitochondrial Function Impairs Lysosomes. J Biol Chem. 2016; 291:10263–76. 10.1074/jbc.M115.695825 26987902 PMC4858975

[27] Kimura S, Noda T, Yoshimori T. Dissection of the autophagosome maturation process by a novel reporter protein, tandem fluorescent-tagged LC3. Autophagy. 2007; 3:452–60. 10.4161/auto.4451 17534139

[28] Galluzzi L, Pietrocola F, Bravo-San Pedro JM, Amaravadi RK, Baehrecke EH, Cecconi F, Codogno P, Debnath J, Gewirtz DA, Karantza V, Kimmelman A, Kumar S, Levine B, et al. Autophagy in malignant transformation and cancer progression. EMBO J. 2015; 34:856–80. 10.15252/embj.201490784 25712477 PMC4388596

[29] Betz C, Stracka D, Prescianotto-Baschong C, Frieden M, Demaurex N, Hall MN. Feature Article: mTOR complex 2-Akt signaling at mitochondria-associated endoplasmic reticulum membranes (MAM) regulates mitochondrial physiology. Proc Natl Acad Sci USA. 2013; 110:12526–34. 10.1073/pnas.1302455110 23852728 PMC3732980

[30] Ramanathan A, Schreiber SL. Direct control of mitochondrial function by mTOR. Proc Natl Acad Sci USA. 2009; 106:22229–32. 10.1073/pnas.0912074106 20080789 PMC2796909

[31] Morita M, Prudent J, Basu K, Goyon V, Katsumura S, Hulea L, Pearl D, Siddiqui N, Strack S, McGuirk S, St-Pierre J, Larsson O, Topisirovic I, et al. mTOR Controls Mitochondrial Dynamics and Cell Survival via MTFP1. Mol Cell. 2017; 67:922–935.e5. 10.1016/j.molcel.2017.08.013 28918902

[32] Correia-Melo C, Marques FD, Anderson R, Hewitt G, Hewitt R, Cole J, Carroll BM, Miwa S, Birch J, Merz A, Rushton MD, Charles M, Jurk D, et al. Mitochondria are required for pro-ageing features of the senescent phenotype. EMBO J. 2016; 35:724–42. 10.15252/embj.201592862 26848154 PMC4818766

[33] Rabinovitch RC, Samborska B, Faubert B, Ma EH, Gravel SP, Andrzejewski S, Raissi TC, Pause A, St-Pierre J, Jones RG. AMPK Maintains Cellular Metabolic Homeostasis through Regulation of Mitochondrial Reactive Oxygen Species. Cell Reports. 2017; 21:1–9. 10.1016/j.celrep.2017.09.026 28978464

[34] Baba M, Takeshige K, Baba N, Ohsumi Y. Ultrastructural analysis of the autophagic process in yeast: detection of autophagosomes and their characterization. J Cell Biol. 1994; 124:903–13. 10.1083/jcb.124.6.903 8132712 PMC2119983

[35] Szalai P, Hagen LK, Sætre F, Luhr M, Sponheim M, Øverbye A, Mills IG, Seglen PO, Engedal N. Autophagic bulk sequestration of cytosolic cargo is independent of LC3, but requires GABARAPs. Exp Cell Res. 2015; 333:21–38. 10.1016/j.yexcr.2015.02.003 25684710

[36] Mukhopadhyay S, Saqcena M, Chatterjee A, Garcia A, Frias MA, Foster DA. Reciprocal regulation of AMP-activated protein kinase and phospholipase D. J Biol Chem. 2015; 290:6986–93. 10.1074/jbc.M114.622571 25632961 PMC4358122

[37] Rabanal-Ruiz Y, Korolchuk VI. mTORC1 and Nutrient Homeostasis: The Central Role of the Lysosome. Int J Mol Sci. 2018; 19:818. 10.3390/ijms19030818 29534520 PMC5877679

[38] Settembre C, Zoncu R, Medina DL, Vetrini F, Erdin S, Erdin S, Huynh T, Ferron M, Karsenty G, Vellard MC, Facchinetti V, Sabatini DM, Ballabio A. A lysosome-to-nucleus signalling mechanism senses and regulates the lysosome via mTOR and TFEB. EMBO J. 2012; 31:1095–108. 10.1038/emboj.2012.32 22343943 PMC3298007

[39] Settembre C, Di Malta C, Polito VA, Garcia Arencibia M, Vetrini F, Erdin S, Erdin SU, Huynh T, Medina D, Colella P, Sardiello M, Rubinsztein DC, Ballabio A. TFEB links autophagy to lysosomal biogenesis. Science. 2011; 332:1429–33. 10.1126/science.1204592 21617040 PMC3638014

[40] Nelyudova A, Aksenov N, Pospelov V, Pospelova T. By blocking apoptosis, Bcl-2 in p38-dependent manner promotes cell cycle arrest and accelerated senescence after DNA damage and serum withdrawal. Cell Cycle. 2007; 6:2171–77. 10.4161/cc.6.17.4610 17882791

[41] Dimri GP, Lee X, Basile G, Acosta M, Scott G, Roskelley C, Medrano EE, Linskens M, Rubelj I, Pereira-Smith O, Peacocke M, Campisi J. A biomarker that identifies senescent human cells in culture and in aging skin in vivo. Proc Natl Acad Sci USA. 1995; 92:9363–67. 10.1073/pnas.92.20.9363 7568133 PMC40985

