# Supplementary material for: Suppression of mTORC1 activity in senescent Ras-transformed cells neither restores autophagy nor abrogates apoptotic death caused by inhibition of MEK/ERK kinases
Source: Aging (Albany NY). 2018 Nov 27;10(11):3574–89. doi: 10.18632/aging.101686 (PMC6286832; doi:10.18632/aging.101686)
Supplement: Supplementary Figure [file aging-10-101686-s001.pdf]

SUPPLEMENTARY FIGURE

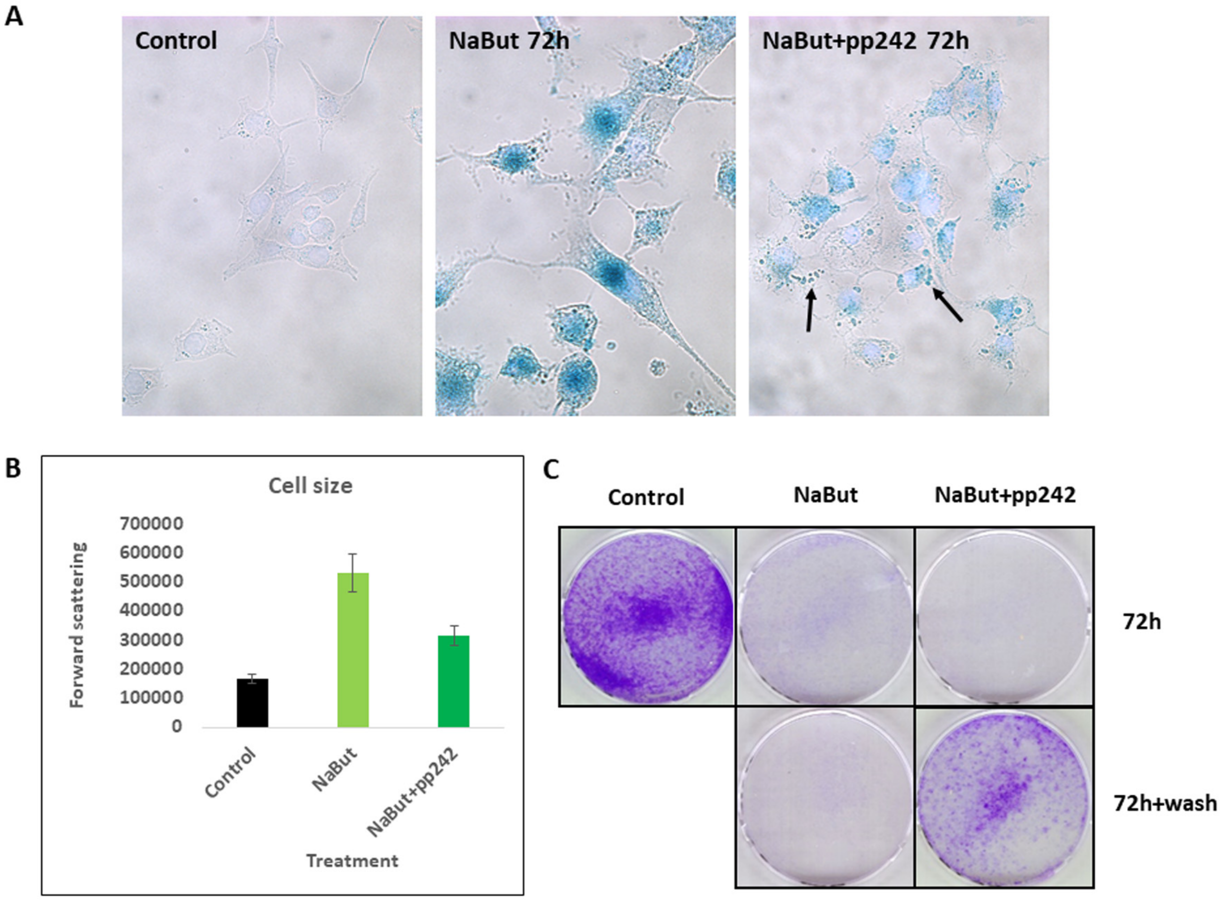

Supplementary Figure1. mTOR suppression with 200 nM pp242 weakens development of senescence phenotype but does not completely decelerate it. (**A**) Activity of senescence-associated β-galactosidase in senescent cells with active mTOR and after 72 h mTORC1 suppression. The pictures were taken by using Pascal LSM 5 microscope. (**B**) The size of senescent cells treated with pp242 for 72 h. Histograms represent forward scattering obtained by flow cytometry. (**C**) Regrowth analysis depicting cellular ability of pp242 to restore proliferation of senescent cells. Cells were treated with inhibitors for 72 h, then washed and cultivated in the medium without inhibitors for 48 h. Staining with Crystal Violet.
